# Gene therapy for deafness: are we there now?

**DOI:** 10.1038/s44321-024-00058-6

**Published:** 2024-03-25

**Authors:** Tobias Moser, Han Chen, Kathrin Kusch, Rüdiger Behr, Barbara Vona

**Affiliations:** 1 Institute for Auditory Neuroscience and InnerEarLab, University Medical Center Göttingen, 37099 Göttingen, Germany; 2 Auditory Neuroscience and Synaptic Nanophysiology Group, Max-Planck-Institute for Multidisciplinary Sciences, Göttingen, Germany; 3 Auditory Neuroscience and Optogenetics Laboratory, German Primate Center, Göttingen, Germany; 4 Cluster of Excellence “Multiscale Bioimaging: from Molecular Machines to Networks of Excitable Cells” (MBExC), University of Göttingen, Göttingen, Germany; 5 Functional Auditory Genomics group, Auditory Neuroscience and Optogenetics Laboratory, German Primate Center, Göttingen, Germany; 6 Platform Degenerative Diseases, German Primate Center, Göttingen, Germany; 7 Institute of Human Genetics, University Medical Center Göttingen, 37099 Göttingen, Germany

**Keywords:** Genetics, Gene Therapy & Genetic Disease

## Abstract

This Commentary discusses the successes of the recent first in human trials for gene therapy of otoferlin-deficient hearing impairment.

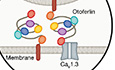

## Teaser

Approximately half a billion people—5% of the world’s population—suffer from disabling hearing impairment (HI) according to the WHO (http://www.who.int/features/factfiles/deafness/en/). HI commonly hampers speech acquisition, leads to social isolation and increases risk for depression and cognitive decline. One to two per thousand children are born with disabling HI and over 50% of sensorineural HI is caused by defects in individual genes (deafness genes) of which roughly 150 have been identified so far (http://hereditaryhearingloss.org/). In case of profound hearing impairment or deafness, cochlear implants that bypass the dysfunctional or lost sensory hair cells and electrically stimulate the auditory nerve partially restore hearing. However, hearing with cochlear implants has shortcomings such as limited understanding of speech in background noise. So, there remains a major unmet medical need for improved hearing restoration (Wolf et al, 2022). Yet, despite major research efforts, a causal treatment based on pharmacology, gene therapy, or stem cells had, so far, not been clinically available. Now, this is finally changing at least for some patients: first in human trials prove the concept for inner ear gene therapy of otoferlin-related synaptic deafness.

**Figure 1:**
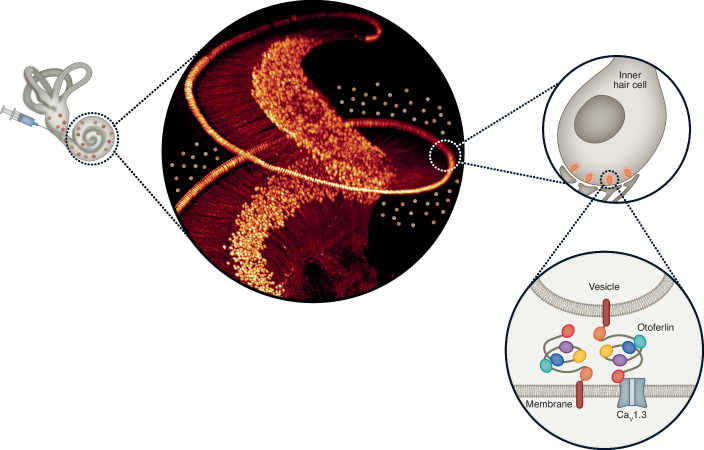
Gene therapy of genetic deafness. Left: schematic drawing of the administration of the gene therapy medicinal product (adeno-associated virus [AAV] carrying the coding sequence for *OTOF*) to the inner ear consisting of the snail-shaped cochlea and the vestibular apparatus. Middle: 3D rendering of the mouse cochlea from light sheet microscopy with viral particles (not to scale, image taken from (Michanski et al, 2019)). Right: schematic drawing of the target cell: the inner hair cell that uses ribbon synapses to signal sound information to the auditory nerve which strictly depends on otoferlin’s role in Ca^2+^ triggered fusion of synaptic vesicles to the active zone plasma membrane (lower image taken from (Chen et al, 2023)).

Only about 5 years after the first preclinical proof of concept for gene therapy of otoferlin-related auditory synaptopathy (Al-Moyed et al, 2018; Akil et al, 2019), preliminary reports from several clinical trials (NCT05901480, ChiCTR2200063181, NCT05821959, NCT05788536) indicate safety and efficacy. These first in human trials of gene replacement therapy are focusing on deafness arising from mutations in the *OTOF* gene (Yasunaga et al, 1999). The choice resulted from the prevalence (up to ~7% of hereditary non-syndromic HI) and largely maintained structure of the sensory organ offering a therapeutic window for gene therapy. *OTOF* codes for the hair cell synaptic protein otoferlin (Fig. 1) and mutations cause autosomal recessive HI ranging from mild threshold increases (some with temperature-dependent worsening) to deafness (Moser and Starr, 2016*)*. This HI results from defective sound encoding at the synapse between inner hair cells and auditory nerve fibers: auditory synaptopathy (Roux et al, 2006). It has become clear that otoferlin has multiple functions in Ca^2+^ triggered release and replenishment of synaptic vesicles (Moser and Starr, 2016). The latter has the most stringent requirement on the abundance of the protein. Given the high rate of transmission at the hair cell synapse, efficient vesicle replenishment is strictly required for coding temporally structured sound stimuli such as speech.

This suggests a stringent need on exogenous otoferlin expression in gene replacement. The need to deal with a large coding sequence exceeding the packaging capacity of adeno-associated virus (AAV), standard gene therapy vectors, has added further challenges. Preclinical trials of *OTOF* gene therapy in mice using the dual-AAV or overloaded AAV (Al-Moyed et al, 2018; Akil et al, 2019; Rankovic et al, 2021) had demonstrated partial recovery of hearing. Vesicle replenishment remained below the normal speed in transduced hair cells which most likely reflected that exogenous otoferlin expression did not fully achieve control levels (Al-Moyed et al, 2018). Therefore, one of the remaining worries has been that the efficacy of clinical gene therapy could also be limited and that this would be detected late as a slow and potentially incomplete speech development. Moreover, applying the AAV gene therapy medicinal product (GTMP) to the cochlea (Fig. 1) without damaging the function of the delicate inner ear is far from trivial. Therefore, translating gene therapy for otoferlin-related auditory synaptopathy has faced many challenges.

Academic and private sector programs have undertaken massive efforts to bring gene therapy into clinical trials during the past years. These days, we witness the first reports on small numbers of children dosed with dual-AAV that describe proof of concept for clinical gene replacement therapy of the cochlea (Qi et al, 2024; Lv et al, 2024). So far, no dose-limiting toxicity or serious adverse events have been observed, which is great news also for developing cochlear gene therapy beyond otoferlin-related auditory synaptopathy. Hearing thresholds assessed by recordings of auditory brainstem responses and psychophysical pure tone audiometry improved from not measurable to near normal ~30 dB (hearing level). This is truly remarkable, because auditory brainstem responses are very sensitive to even subtle alterations of synaptic sound encoding. They rely on synchronized activation of many spiral ganglion neurons, which in turn builds on high rates of glutamate release at their ribbon synapses with inner hair cells. In fact, poor or lost auditory brainstem responses despite preserved cochlear function upstream of the inner hair cells is the diagnostic signature of an auditory synaptopathy (Moser and Starr, 2016). Moreover, speech recognition was reported in children with improved hearing. Impressively, the age range of children, for whom published data on hearing restoration upon gene therapy exist, is quite broad 1–8 years, indicating that the therapeutic window for cochlear repair is relatively long. All this is very exciting and the field is anxiously waiting for data to come along regarding speech development and speech recognition if dosed before or within the critical window of speech development or in children who had undergone cochlear implantation before the dosing. Further important questions include (i) How reliable is hearing restoration by *OTOF* gene therapy, (ii) Is the current implementation of *OTOF* gene therapy suitable for all patients with otoferlin-related auditory synaptopathy, and might there be differences in optimal dose? (iii) How stable is hearing restoration by *OTOF* gene therapy? (iv) How do the efficacy and stability compare for different capsid-promotor combinations? (v) What is the optimal immunomodulation protocol? (vi) What is the most suitable regimen for treating both ears? The relevance of the first two questions is highlighted by the finding of a non-responder in one of the first studies (Lv et al, 2024). Lessons learned from ocular gene therapy of Leber’s congenital amaurosis type II with a follow-up beyond 9 years, indicate that the treatment overall is reliable as improvement of vision outlasts several years and that the dosing of the other eye is safe (Russell et al, 2017).
